# Miss Matheson's Address

**Published:** 1917-11-10

**Authors:** 


### NURSING AFFAIRS IN IRELAND.

### Miss Matheson's Address.

MAY I be permitted to offer for the consideration of your readers some comments on the address published in your last issue by Miss Matheson, Secretary to the Irish Board of the College of Nursing? Miss Matheson is a scholar, a mathematical scholar at that, having captured honours of a particularly distinguished character at Dublin University before she adopted nursing as a career. Anything that she says, therefore, is almost bound to be, before it is uttered, logical and conclusive. Her words are those of a nurse, trained and experienced, addressed to her sisters in the profession, and they are, in addition, obviously and necessarily the voice of her Board, the message of the Irish Branch of the College of Nursing to all whom it may concern in this country.

Having paid this tribute to Miss Matheson's address, I have to confess to a feeling of disappointment, not so much with anything that was said, but with what was left unsaid on the occasion, and therefore I propose, with your consent, to offer some constructive and, I hope, helpful criticism. I think that this criticism is urgently necessary at the moment, and I am not sure that it does not apply quite as much to Headquarters' policy as to that which is purely Irish.

### THE BREAD-AND-BUTTER ARGUMENT.

To begin with, Miss Matheson's address conveys the impression that her Board is patiently waiting, with an apparently faultless organisation, for an accession of recruits, and the lecturer informed her hearers that already 7,000 nurses had enrolled. "Let us sweep away the cobwebs which stretch across our views," said Miss Matheson in concluding, "and see clearly the goal and the path to that goal." The goal is a thoroughly well organised nursing profession, formed of a body of fully-educated, fully-trained women, with high ideals, accurate knowledge, and skilled touch, co-operating in the wise management of their profession and the perpetuation of its highest ideals and standards. The path to that goal is an organisation which will make possible all these things and which, by embracing the whole of the profession, will enable nurses to be fully trained, fully educated, and adequately paid, and will give to every nurse the opportunities to work along the line of her profession which most appeals to her." This is all very beautiful and doubtless very true, but altogether too vague and too general. In Ireland the nurse has very pressing grievances which need remedying, and the only scheme which is likely to appeal to her is one which affords her convincing prooof that practical steps are actually being taken to bring her relief. There is the hospital nurse, who spends four and a half years with little or no salary earning her diploma, and who pays a large entrance fee in order to enable her to achieve even this. There is the Poor-Law nurse, constantly engaged in battling, all unaided, with Poor-Law Guardians and Local Government regulations. There is the district nurse, who subsists on the fruits of charity bazaars and sugar-cake raffles. There is the private nurse, segregated from the remainder of her kind, eking out a hand-to-mouth existence. Not one of these has a pension to look forward to in retirement, nor a benevolent fund to draw upon in sickness. These are merely one or two of the more outstanding concerns of Irish nurses, and if the College is to be a force in this country, such material considerations must be taken in hand with boldness and determination. High ideals and the alleviation of the suffering of humanity represent only half of the truth. A man becomes a doctor doubtless with all this in view, but what he is primarily in search of is a career, and likewise the object of a barrister is not, in the first instance, the pure administration of justice. Is it to be expected that altruism and philanthropy are to be regarded as the guiding star of the numberless women who enter the nursing profession? Is the bread-and-butter argument a matter of no consequence?

### NURSING AND TRADES UNIONISM.

It appears to be recognised on every side that nursing should on no account ally itself with trades unionism. It would, for example, be unthinkable that any Union of Nurses could call a strike and leave suffering humanity to die whilst its members fought for a wage benefit. It is possible, however, while endeavouring to avoid Scylla, to be engulfed in Charybdis. The material concerns of the nursing profession are of paramount importance, and, with great respect, I submit for the consideration of the College authorities that there is no evidence to show that vigorous action concerning any or all of these matters has even been considered. The College authorities should profit by the mistakes of the semi-defunct Irish Nurses' Association, which sat for twenty years or thereabouts with folded hands, awaiting the coming of State Registration. No Irish nurse now takes any interest in its deliberations, and for the elementary reason that they are purely academic.

### WHAT IRISH NURSES WANT.

Much is made of the political atmosphere as it affects or may affect nurses in forming this or that association, and at present there is no ignoring that noxious influences are at work in order to secure their alliance. This, however, is purely superficial. In her heart of hearts every nurse realises that her work and her profession are strictly neutral, and also that her needs are of a material kind. If I may suggest it, the College propaganda ought to begin without delay. A section of its activity ought to be concerned with legislation of every kind affecting the profession. Other sections ought to be given up to an investigation of the wants of the hospital nurse, the district nurse, the Poor-Law nurse, and the hostel system ought to be extended for the betterment of the private nurse. When the individuals of the profession realise that these matters constitute the main business of the Irish Board of the College of Nursing, membership will follow as a matter of course.

IERNE.

